# Application of Volatile Organic Compound Analysis in a Nutritional Intervention Study: Differential Responses during Five Hours Following Consumption of a High‐ and a Low‐Fat Dairy Drink

**DOI:** 10.1002/mnfr.201900189

**Published:** 2019-08-05

**Authors:** Jeske H. J. Hageman, Arie G. Nieuwenhuizen, Saskia M. van Ruth, Jos A. Hageman, Jaap Keijer

**Affiliations:** ^1^ Human and Animal Physiology Wageningen University 6708 WD Wageningen The Netherlands; ^2^ FrieslandCampina 3818 LE Amersfoort The Netherlands; ^3^ Food Quality and Design/RIKILT Wageningen University and Research 6700 AE Wageningen The Netherlands; ^4^ Biometris Wageningen University and Research 6700 AA Wageningen The Netherlands

**Keywords:** breath analysis, breathomics, inter‐ and intraindividual variation, lipids, volatile organic compounds (VOCs)

## Abstract

**Scope:**

Exhaled volatile organic compounds (VOCs) are a possible relevant target for noninvasive assessment of metabolic responses. Using a breathomics approach, it is aimed to explore whether lipid intake influences VOC profiles in exhaled air, and to obtain insight in intra‐ and interindividual variations.

**Methods and results:**

Three human interventions are performed. In the first, 12 males consume a high‐fat drink on three study days. In the second, 12 males receive a high‐ and a low‐fat drink on 6 days. In the third, three volunteers consume the high‐fat drink again for tentative compound identification. Participants are asked to exhale, for 5 h postprandial with 15–20 min intervals, into a proton‐transfer‐reaction mass spectrometer, and VOCs in exhaled air are measured. Consumption of a drink alters the VOC profile, with considerable interindividual variation and quantitative intraindividual differences between days. Consumption of two different drinks results in a distinct VOC profile, caused by several specific *m*/*z* values. Most of these compounds are identified as being related to ketone body formation and lipid oxidation, showing an increase in high‐ versus low‐fat drink.

**Conclusion:**

Exhaled VOCs have the potential to assess differences in metabolic responses induced by nutrition, especially when day‐to‐day variation can be minimized.

## Graphical abstract

**Exhaled air** contains volatile organic compounds (VOCs), which are partly derived from the body's internal metabolism. It is shown that differences in VOC profiles can be identified after consumption of dairy drinks differing in amount of lipids. This indicates that analysis of exhaled VOCs may be a useful method to study metabolic effects of nutrition.

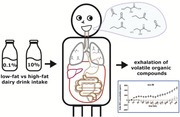

## 1 Introduction

Currently, effects of nutrition or specific food components on absorption or metabolism are mostly studied via biomarkers measured in sampled blood. However, in specific target groups like infants, children, or elderly, this invasive method is not always desirable. To reduce the burden on participants of nutritional intervention studies, there is a need for noninvasive assessment methods. Analysis of exhaled compounds, breathomics, may be such an interesting noninvasive method. With each exhaled breath, a mixture of molecules is released into the air.^1^ Some of these molecules are volatile organic compounds (VOCs), which are partly derived from the body's internal metabolism.^2^ Approximately 200 VOCs are present in a breath sample analyzed with GC‐MS, which differ per individual.^3^ VOCs in exhaled air have been related to volatile components present in blood, since several studies show similar VOCs being present in blood and in exhaled air, in similar concentrations.^4^, ^5^ Analysis of the composition of the VOCs present in exhaled air can, therefore, give an indication about the physiological processes that occur in the body.^1^, ^2^, ^6^ Diseases and health status have been shown to influence the profile of VOCs in exhaled air.^6^, ^7^, ^8^ Specific examples suggest that VOC patterns in exhaled air indeed may reveal metabolic pathways impacted by nutrition. For instance, during fasting, acetone is measured in exhaled air,^9^ which is likely to reflect increased ketogenesis. Furthermore, a recent study shows that consumption of two different infant formulas results in a different postprandial VOC pattern.^10^

The use of VOCs analysis to assess direct effects of nutrition on metabolism comes with some challenges. One of those is the qualitative and quantitative interindividual and intraindividual variation in the VOCs in exhaled air.^11^ Concentrations of VOCs may vary considerably between individuals,^3^ for example, due to smoking, gender, age, and BMI.^11^, ^12^ The intraindividual variation has received little attention so far. Changes over time within subjects over several exhalations,^13^ several hours,^14^ or several days^15^ have been reported, but, to our knowledge, day‐to‐day variation in response to a meal has not been investigated.

The suitability of VOCs analysis to study metabolic responses in a nutritional intervention study is depending on the magnitude of effects of a nutritional challenge on VOCs, relative to the interindividual, and, more importantly, the intraindividual variation in VOCs in exhaled air. One objective of this study was therefore to study intra‐subject variation in VOCs in exhaled air after a nutritional intervention. Since it should also be possible to discriminate between nutritional interventions, another objective was to establish whether the consumption of a high‐fat versus low‐fat dairy drink resulted in a different VOC profile in exhaled air. Furthermore, we aimed to tentatively identify affected VOCs for insight in potentially affected metabolic pathways.

## 2 Experimental Section

### 2.1 Subjects

Both for intervention 1 and 2, 12 healthy male volunteers (intervention 1: 22.8 ± 3.0 years of age, intervention 2: 22.5 ± 2.7 years of age) from Wageningen and surroundings were enrolled. To avoid confounding factors such as differences in gender, age, and BMI,^12^ only healthy males aged 18–35 years, with a BMI between 22 and 25 kg m^−2^ and with a relative body fat mass between 8% and 15% were included. Smokers, or subjects who smoked in the past, were excluded. The main subject characteristics are presented in **Table** 1. Informed consent was obtained from the volunteers before their involvement in the intervention. The Wageningen University ethics committee (METC‐WU) (NL56722.081.16) approved this study protocol. The study (Breath Taking) was registered at the NTR (NTR5974). The study was conducted in accordance with the principles of the Declaration of Helsinki (Fortaleza, Brazil 2013).

**Table 1:** Subject characteristics (*n* = 12) (mean ± SD)

	Intervention 1	Intervention 2
Age [years]	22.8 ± 3.0	22.5 ± 2.7
Body weight [kg]	79.6 ± 6.7	74.8 ± 6.4
BMI [kg m^−2^]	23.4 ± 1.3	22.4 ± 1.0
Fat mass [%]	13.0 ± 2.2	12.3 ± 2.2

### 2.2 Methods

#### 2.2.1 Intervention 1

Subjects were restricted from high‐intensity exercise, alcohol consumption, and usage of recreational drugs and medication the day before a study day. The evening preceding a study day, the subjects consumed a standardized dinner (see **Table** 2) followed by a 12 h overnight fast. The next morning subjects consumed 500 mL of a high‐fat dairy drink (see **Table** 3) within a period of 5 min. Exhaled air was measured before consumption (baseline) and every 15 min after consumption until 5 h postprandial. The subjects were asked to breath into a mouthpiece connected to a breath sampling device (Buffered End‐Tidal breath sampling inlet, Ionicon Analytik G.m.b.H., Innsbruck, Austria),^16^ which was connected to a proton transfer reaction‐mass spectrometer (PTR‐MS) (Ionicon Analytik G.m.b.H., Innsbruck, Austria) for immediate and online analysis of breath samples. Mass‐to‐charge ratios (*m*/*z*) of *m*/*z* 21–160 were determined, with a dwell time of 0.1 s mass^−1^, at a drift pressure of 2.20 mbar, and chamber and inlet temperature of 60 °C. Per time point three replicate analyses of ten cycles were performed. In between measurements, the volunteers were asked to keep in a seated, rested position. Ambient air was analyzed in between all measurements to determine whether any fluctuations over time occurred. This procedure was repeated three times, on different days with at least one day of wash‐out in between.

**Table 2:** Composition of the standardized dinner

Per portion	Standardized dinner
Energy (kcal)	876
Protein [g]	33.3
Fat [g]	37.0
Carbohydrates [g]	94.9

**Table 3:** Composition of the dairy drinks

Per 100 mL	High‐fat dairy drink	Low‐fat dairy drink
Energy (kcal)	124.8	36.5
Protein [g]	3.7	3.7
Fat [g]	9.9	0.1
Carbohydrates [g]	5.2	5.2

Protein, carbohydrate, and fat sources were identical in both products, different amount of fats were added to the drinks resulting in a high‐fat and low‐fat dairy drink with different energy contents.

#### 2.2.2 Intervention 2

In the second intervention, the same procedure as described above for intervention 1 was followed, except that two different nutritional interventions were applied: one consisting of a high‐fat dairy drink and the other of a low‐fat dairy drink (Table 3). In a cross‐over design, participants received the low‐fat and high‐fat dairy drink three times each, all in randomized order, on six different study days, which were separated by at least one day of wash‐out.

#### 2.2.3 Intervention 3

To be able to tentatively identify specific VOCs related to the intake of the dairy drinks, three participants of the first two interventions were invited for another study day. The same procedure as that of the previous interventions with the consumption of the same high‐fat dairy drink was used, except that breath measurements were performed every 20 min postprandial, and the breath sampling device was coupled to a PTR‐Quadrupole interface Time of Flight‐MS (PTR‐QiToF‐MS) (Ionicon Analytik G.m.b.H., Innsbruck, Austria), which provides a more accurate mass to enable tentative compound identification. Ionization of H_3_O^+^ was performed under drift tube voltage of 900 V, and an inlet temperature of 60 °C. Mass‐to‐charge ratios (*m*/*z*) of *m*/*z* 17.002 to *m*/*z* 445.123 were determined in all exhalations, with a dwell time of 0.4 s mass^−1^.

#### 2.2.4 VOCs in Dairy Drink

To tentatively identify the VOCs present in the dairy drinks, the drinks were transferred to a glass bottle with a mechanical stirrer and the headspaces of the low‐fat (*n* = 2) as well as the high‐fat dairy drink (*n* = 4) were measured using PTR‐MS (Ionicon Analytik G.m.b.H., Innsbruck, Austria). Mass‐to‐charge ratios (*m*/*z*) of mass range *m*/*z* 21–160 were determined in five cycles, with a dwell time of 0.1 s mass^−1^, at a drift pressure of 2.20 mbar, inlet flow of 54 mL min^−1^, and chamber and inlet temperature of 60 °C.

### 2.3 Data Preprocessing

Prior to statistical analysis of the data of interventions 1 and 2, several steps of preprocessing were performed. For these studies, the raw counts were converted into ppbv (parts per billion by volume). *m*/*z* 32 (O2+) and *m*/*z* 37 (water cluster ion) associated with the PTR‐MS ion source were removed from the dataset. The correlation of the different replicates per time point was checked and uncorrelated cycles were removed. The analytical level of detection (LOD) of the PTR‐MS was determined as three times the signal‐to‐noise ratio of blank *m*/*z* values in 4350 ambient air samples, which was 0.187 ppbv. Denoising was performed by replacing all concentrations below 0.2 ppbv (i.e., slightly above the analytical LOD) with 0.2 ppbv and the *m*/*z* values with 80% or more of the measurements being 0.2 ppbv were removed from the dataset. Prior to principal component analysis (PCA), *m*/*z* values were logtransformed and scaled. To remove variation caused by subjects, the *m*/*z* values were corrected for baseline values and meancentered per subject. To obtain a global understanding of the variation in the *m*/*z* values of intervention 2 related to the time points and the two treatments, baseline corrected *m*/*z* values were averaged across the different participants for the two treatments separately. PTRwid was used to execute mass scale calibration and peak extraction of the data of intervention 3, gathered via PTR‐QiToF‐MS.^17^

### 2.4 Statistics

#### 2.4.1 Intervention 1

A PCA of the data of intervention 1 was performed and a PCA score plot was created in Rstudio (version 1.1.456 for Windows, RStudio Inc.) using R (version 3.5.1 for Windows) and package FactoMineR, to investigate the differences between participants (uncorrected for baseline and without meancentering per subject), the effect of the meal, and the effect of study days. For each *m*/*z* value, net area under the curve (AUC) was calculated using GraphPad Prism (version 5.04 for Windows, GraphPad Software, San Diego, CA, USA), and the different study days were compared using repeated measures ANOVA. A *p*‐value <0.05 was considered to be significantly different.

#### 2.4.2 Intervention 2

For each *m*/*z* value, net area under the curve (AUC) was calculated using GraphPad Prism (version 5.04 for Windows, GraphPad Software, San Diego CA, USA), and the two dairy drinks were compared using paired *t*‐test. A *p*‐value <0.05 was considered to be significant different. PCA was used to obtain a low dimensional summary of all *m*/*z* values for the two treatments and time points. The change over time of each *m*/*z* value was modeled using a segmented linear regression for each participant and measurement day separately, using the R‐package segmented.^18^ Segmented regression allowed for modeling each *m*/*z* curve by two individual linear regression lines connected by a flexible breakpoint. This allowed to mimic a steady change in intensity followed by a period of constant intensity. This way, each *m*/*z* curve was summarized by two intercepts, two slopes, and a breakpoint. For each *m*/*z* value and drink, the change in intensity in time was plotted using the participant and measurement day averaged data. Super positioned in this overview are the averaged segmented regression models. The effect of the different drinks was tested on the two different intercepts and two different slopes with the following mixed model:

$$\mathrm{response}∼\mathrm{TypeDrink}+\left(1|\mathrm{MeasurementDay}/\mathrm{Participant}\right)$$

where *TypeDrink* was the fixed treatment effect and *(1| MeasurementDay / Participant)* was a random term taking into account that participants were nested within measurement days. A *p*‐value <0.05 was considered to be significantly different.

#### 2.4.3 Intervention 3

The data of this intervention was used to provide responses with a more accurate *m*/*z* values to enable tentative compound identification. A profile comparison was performed of those more accurate *m*/*z* values and the *m*/*z* values that showed a different response over time between the two drinks in intervention 2. Thereafter, ChemCalc was used to obtain corresponding molecular formulas,^19^ and the Human Metabolome Database (HMDB) and literature was used to derive possible compounds from these formulas.^20^

#### 2.4.4 VOCs in Dairy Drink

The averages of five cycles were calculated and thereafter averages and standard deviations of the two different dairy drinks were calculated. *m*/*z*‐values with a ppbv above 0.2 were selected and compared. When concentrations of a certain mass differed more than the standard deviation multiplied by two between the two dairy drinks, the concentrations were considered to be different.

## 3 Results

### 3.1 Participants

**Figure 1:**
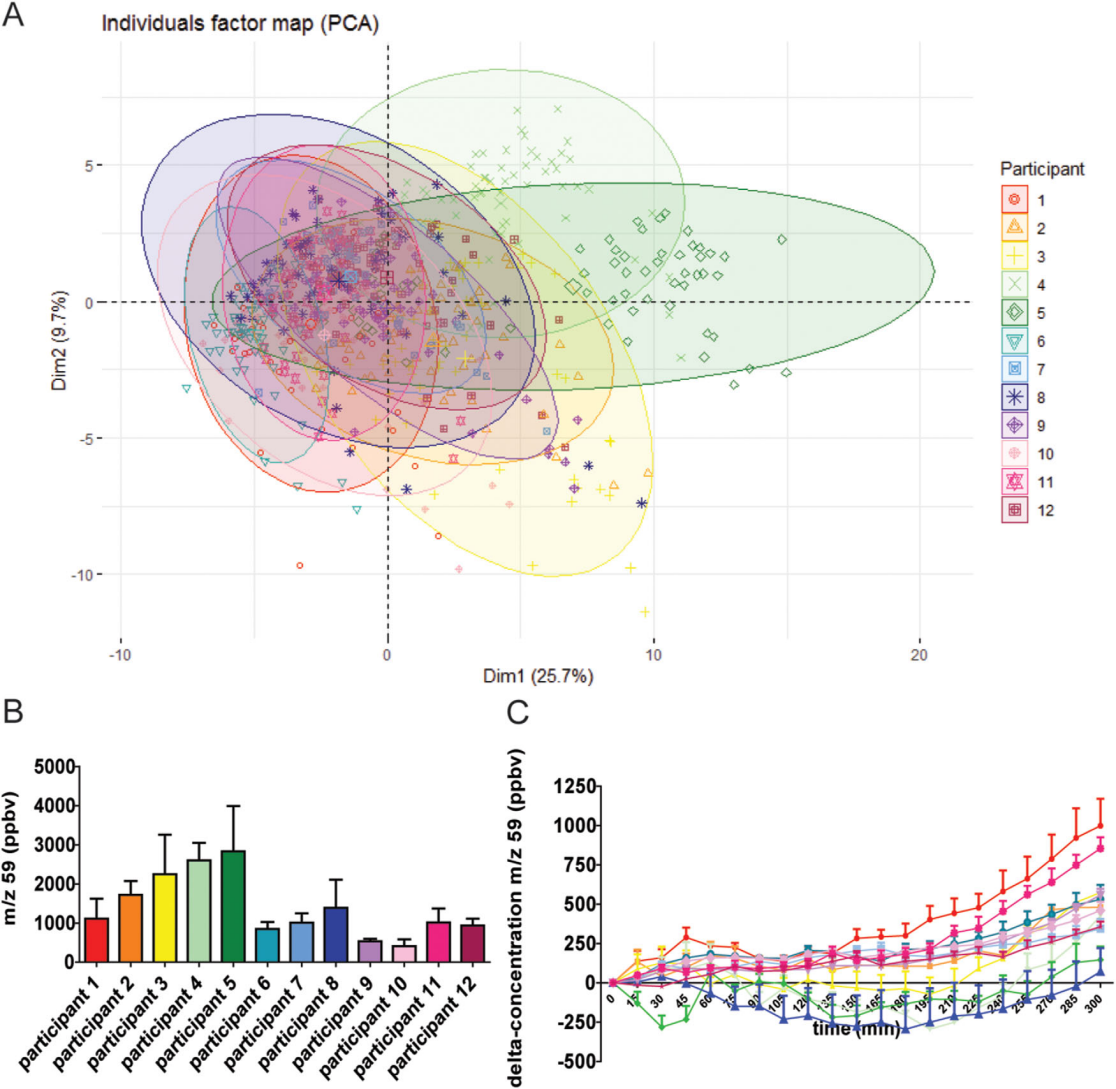
The VOC profiles in exhaled air of different subjects. Subjects were followed over time, VOCs were measured online with PTR‐MS in fasted and postprandial states. A) PCA score plot of 756 breath‐o‐grams performed on 71 VOCs excreted in exhaled air from 12 different subjects. Each color represents a different participant. The subjects form separate clusters. (Logtransformed, scaled data from intervention 1.) B) Absolute concentrations (ppbv) of *m*/*z* 59 in fasted state, average of 3 days (mean ± SD). C) Concentrations of *m*/*z* 59, corrected for baseline concentrations, for the 12 different subjects, average of 3 days (mean ± SD).

Intervention 1 was performed to gain insight in intra‐ and interindividual differences causing variation in VOC profiles. PCA analysis of the VOC concentrations in exhaled air in fasted and postprandial state showed that, despite some overlap, the 12 subjects formed separate clusters based on their VOC profile (**Figure** 1A). In fasted state, the different *m*/*z* values, for example, *m*/*z* 59, differed between subjects (Figure 1B). Although the concentrations after correction for baseline still showed quantitative differences, the trends over time were similar for the participants (Figure 1C).

### 3.2 Variation in Study Days

**Figure 2:**
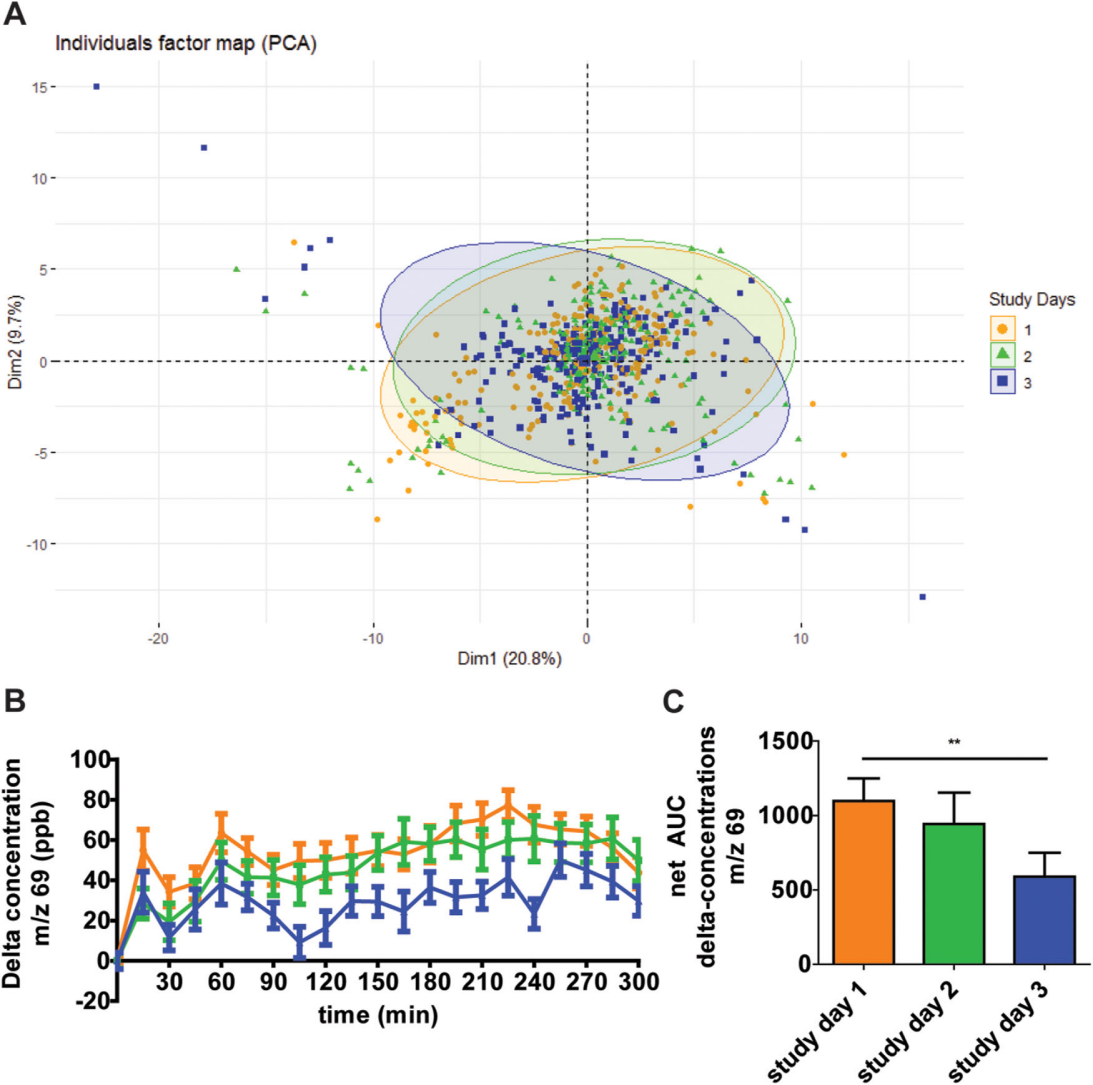
Variation in VOC profile in exhaled air on different study days. A) PCA score plot of 756 breath‐o‐grams on 71 VOCs excreted in breath from 12 different subjects. The different study days are represented, no separate clusters were observed. (Data of intervention 1: cleaned data of all VOCs, corrected for baseline, and meancentered per participant.) B) Concentration of *m*/*z* 69 in exhaled air, corrected for baseline, on the three study days (mean ± SEM). Mean basal concentration (±SEM) was 128.7 ± 8.4. C) Net Area under the Curves (AUC) of the concentrations of *m*/*z* 69 corrected for baseline on the three study days. Statistical analysis was performed using repeated measures ANOVA (*p* = 0.0074), **p < 0.01. The orange symbols, line, and bar represent study day 1, the green symbols represent study day 2, and the blue symbols represent study day 3.

**Figure** 2A shows a PCA plot of the three different study days for each participant. After preprocessing to remove the interindividual variation, the intra‐subject variation was examined by comparing differences between study days. The total VOC profiles on the three study days overlaid each other; no separate clusters were formed. For the *m*/*z* values that changed with time, for example, *m*/*z* 69, a similar qualitative profile after consumption of the high‐fat dairy drink was observed on different study days (Figure 2B). Quantitatively, the three study days resulted in somewhat different net AUCs for some of the components, as is shown for *m*/*z* 69 (*p* = 0.0074) (Figure 2C).

### 3.3 Effect of Nutrition

**Figure 3:**
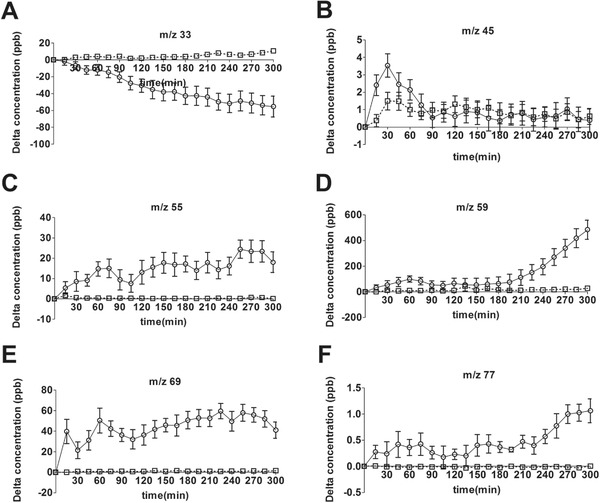
A–F) Effect of a high‐fat drink on the VOC profile in exhaled air. Concentrations of *m*/*z* 33, *m*/*z* 45, *m*/*z* 55, *m*/*z* 59, *m*/*z* 69, and *m*/*z* 77 in exhaled air, corrected for baseline (full line, ○), and ambient air (dotted line, □) (mean ± SEM) (data from intervention 1: mean of 12 participants). Mean basal concentrations (±SEM) were: A) 223.0 ± 28.8, B) 31.3 ± 0.5, C) 107.7 ± 6.4, D) 1392 ± 232.5, E) 128.7 ± 8.4, F) 2.4 ± 0.4.

Besides insight in inter‐ and intra‐subject variation, intervention 1 also showed a time response for some specific *m*/*z* values. **Figure** 3A–F displays specific *m*/*z* values with a different postprandial response. *m*/*z* 33 was found to decrease after consumption of the high‐fat drink. Some VOCs showed a gradual increase over time, such as *m*/*z* 59 or *m*/*z* 77, while others directly increased after consumption, such as *m*/*z* 55 or *m*/*z* 69. Some VOCs, such as *m*/*z* 45, showed a peak in concentration right after consumption of the milk.

### 3.4 Differences Upon the Intake of Different Drinks

**Figure 4:**
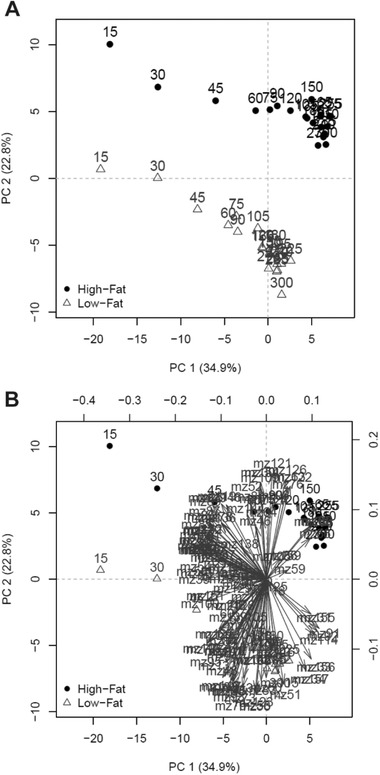
Different effects of high‐fat versus low‐fat dairy drinks on VOC profile in exhaled air. PCA plot of 1486 breath‐o‐grams on 100 VOCs excreted in breath from 12 different subjects A) score plot, B) including loading plot. The black dots (●) represent high‐fat milk, and the grey triangles (∆) represent low‐fat milk. (Data of intervention 2: corrected for baseline and meancentered per participant.)

In intervention 2, two different dairy drinks were provided to study the effect of a difference in lipid intake. **Figure** 4A shows a PCA score plot in which the two drinks formed separate clusters, indicating a difference in VOC profile between the two milk drinks. The two drinks were separated essentially along PC2, which explained almost 23% of the variation in the data. A similar trend in time was visible along the PC1 axis from 15 to 300 min after consumption of the drinks. The corresponding biplot of all *m*/*z* values (Figure 4B) shows which *m*/*z* values were mostly influenced byPC1 (horizontal change) or were mostly affected along PC2 (vertical change).

**Figure 5:**
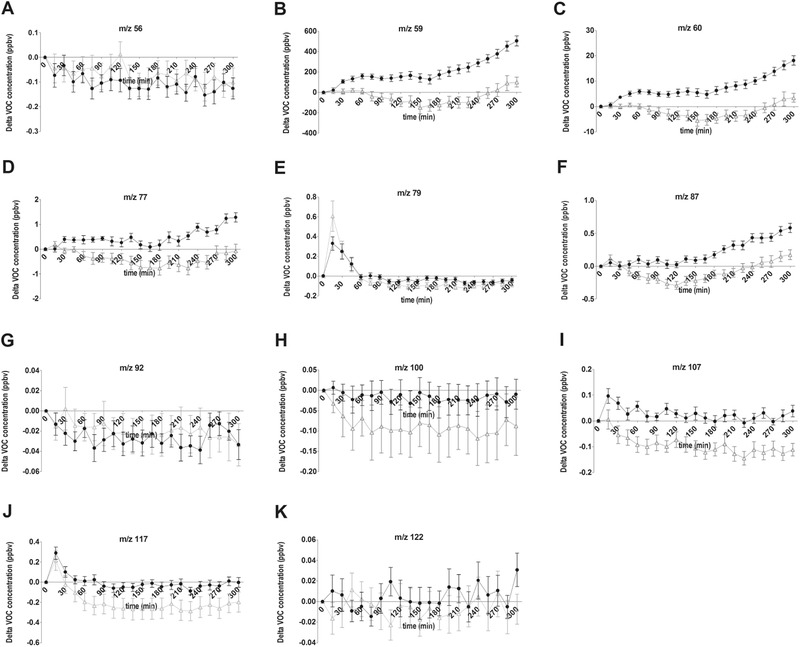
Postprandial VOC *m*/*z* value concentrations, corrected for baseline, in response to high‐fat and low‐fat dairy drink. Concentrations of those *m*/*z* response curves of exhaled VOCs that revealed a difference between the two dairy drinks are shown (mean ± SEM). A) *m*/*z* 56, B) *m*/*z* 59, C) *m*/*z* 60, D) *m*/*z* 77, E) *m*/*z* 79, F) *m*/*z* 87, G) *m*/*z* 92, H) *m*/*z* 100, I) *m*/*z* 107, J) *m*/*z* 117, and K) *m*/*z* 122. The black line (●) represents the high‐fat dairy drink and the grey line (∆) represents the low‐fat dairy drink. (Data from intervention 2: mean of 12 participants). Mean basal concentrations (±SEM) for the low‐fat and high‐fat dairy drink were respectively: A) 0.92 ± 0.06, 0.91 ± 0.05; B) 1141 ± 177.10, 1046 ± 126.6; C) 40.35 ± 6.42, 36.51 ± 4.61; D) 3.35 ± 0.58, 3.00 ± 0.42; E) 0.34 ± 0.03, 0.31 ± 0.02; F) 1.23 ± 0.10, 1.27 ± 0.06; G) 0.25 ± 0.02, 0.25 ± 0.01; H) 0.34 ± 0.07, 0.25 ± 0.04; I) 0.40 ± 0.02, 0.27 ± 0.01; J) 0.60 ± 0.12, 0.41 ± 0.04; K) 0.25 ± 0.01, 0.24 ± 0.01.

To further investigate whether specific *m*/*z* values showed different time responses between the two dairy drinks, segmented regression was used. The segmented regression model summarizes each curve in two intercepts and two slopes. These parameters were used to compare the two dairy drinks using mixed model analysis. **Table** 4 shows the results of this analysis and **Figure** 5 displays the time profiles of selected *m*/*z* values. An overview of all *m*/*z* values is present in Figure S1, Supporting Information.

**Table 4:** *p*‐values of VOC *m*/*z* values where a significant difference in response to the high‐fat versus low‐fat dairy drink was observed for one of the parameters derived from segmented regression analysis, analyzed using a mixed model analysis (*p*‐values below 0.05 were considered to be significant, nonsignificant results are not shown)

VOC	Intercept 1	Slope 1	Intercept 2	Slope 2
*m*/*z* 56		0.013		
*m*/*z* 59		0.009		
*m*/*z* 60		0.005		
*m*/*z* 79			0.046	
*m*/*z* 87		0.009		
*m*/*z* 92			0.009	0.018
*m*/*z* 100			0.007	0.028
*m*/*z* 122		0.047		

**Figure 6:**
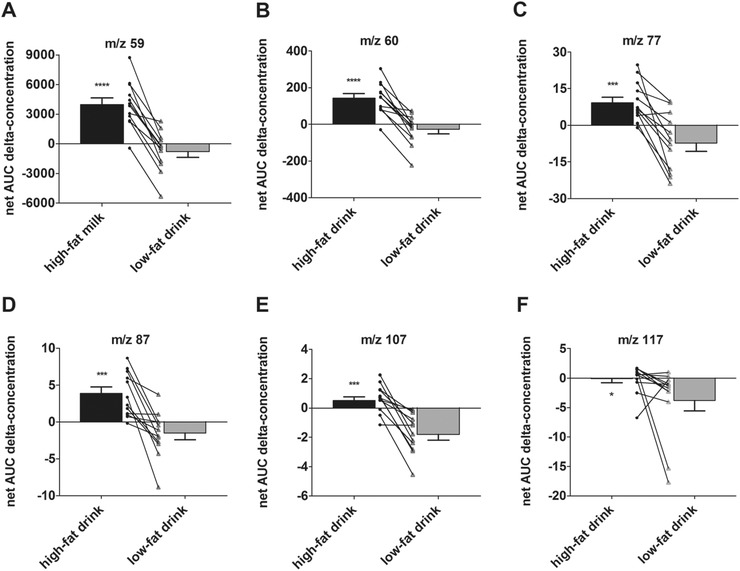
Difference in net AUC of concentrations, corrected for baseline, in exhaled air after two different drinks. Net area under the curve (AUC) (mean ± SEM) and difference in net AUC in response to the high‐fat and low‐fat dairy drink of concentrations, corrected for baseline, of A) *m*/*z* 59, B) *m*/*z* 60, C) *m*/*z* 77, D) *m*/*z* 87, E) *m*/*z* 107, and F) *m*/*z* 117 in exhaled air. The net AUC were compared using a paired *t*‐test or, when the data were not normally distributed, Wilcoxon matched‐pairs signed rank test, *p* < 0.05 was considered to be statistically significant, *p < 0.05, **p < 0.01, ***p < 0.001, ****p < 0.0001. (Data from intervention 2: mean of 12 participants.)

Time responses of 11 *m*/*z* values were different after the consumption of the high‐fat and the low‐fat dairy drink. *m*/*z* 59, *m*/*z* 60, *m*/*z* 77, and *m*/*z* 87 showed a similar time profile. *m*/*z* 59, *m*/*z* 60, and *m*/*z* 87 displayed a first slope of the model that significantly differed between the high‐fat and low‐fat dairy drink, with a positive slope for high‐fat milk and a negative slope for low‐fat milk. Although for *m*/*z* 77 no significant difference between the two drinks was found for the first slope, the two drinks gave a significant different net AUC for this *m*/*z*, similar to *m*/*z* 59, *m*/*z* 60, and *m*/*z* 87 (**Figure** 6). For *m*/*z* 56 and *m*/*z* 92, a slight decrease in time was found. After the consumption of the high‐fat dairy drink, compared to the low‐fat dairy drink, the concentrations of those two compounds were slightly reduced. The first slope of *m*/*z* 56 was significantly different. For *m*/*z* 92, the second intercept and second slope were significantly different between the drinks. Another compound that showed the same differences as *m*/*z* 92, was *m*/*z* 100. However, this compound displayed a different profile in time; the concentrations were less reduced after consumption of the high‐fat drink compared to the low‐fat drink. *m*/*z* 79 and *m*/*z* 117 both showed a peak in concentration at 15 min postprandially. After consumption of the two drinks, the second intercept of *m*/*z* 79 was significantly different. For *m*/*z* 117, the net AUC was significantly higher after consumption of the high‐fat drink compared to the low‐fat drink. A concentration peak was also found for *m*/*z* 107. However, for this compound, a different time response was observed, as the concentration only increased after consumption of the high‐fat drink and after consumption of the low‐fat drink a decrease in concentration was observed. The compound with *m*/*z* 122 displayed a significant different first slope between the time profiles of the two drinks; directly after consumption, the time profiles were inverse for the two dairy drinks.

Figure 6 shows the compounds with an AUC that is significantly different between the low‐fat and the high‐fat drink (*m*/*z* 59, *m*/*z* 60, *m*/*z* 77, *m*/*z* 87, *m*/*z* 107, and *m*/*z* 117). For all six components, a higher net AUC was found after consumption of the high‐fat drink. This pattern was observed for nearly all participants.

### 3.5 Tentative Identification of VOCs

By alignment of the time responses of the *m*/*z* values of intervention 3 to the data of intervention 2, more accurate *m*/*z* values were obtained for those *m*/*z* values that were significant different after consumption of the high‐fat and the low‐fat dairy drink. This was used to determine probable molecular formulas and possible metabolites for these *m*/*z* values (**Table** 5).

**Table 5:** Possible metabolites related to *m*/*z* values differently influenced after consumption of a high‐fat versus a low‐fat dairy drink

*m*/*z*	*m*/*z* (PTR‐QiToF‐MS)	Molecular formula	Potential compounds	References
56	56.044	C_3_H_5_N ° H+	Fragment of heptanal or hexanal	32
59	59.049	C_3_H_6_O ° H+	Acetone	HMDB
60	60.052	^13^C_3_H_6_O ° H+	Isotope of acetone	32
77	77.059	C_3_H_6_O ° H+ + H_2_O	H_2_O cluster of acetone	33
79	79.039	C_6_H_6_ ° H+	Benzene	34
87	87.081	C_5_H_10_O ° H+	2‐Pentanone	28
			2‐Methylbutanal	35
			Pentanal	36
			3‐Methylbutanal (isovaleraldehyde)	37
92	92.049 / 92.059	C_6_H_5_N ° H+	Unknown	
100	100.04	C_4_H_5_NO_2_ ° H+	(R)‐dihydromaleimide NCCOOC2H5 (ethyl cyanoformate) Methyl cyanoacetate 3‐Cyanopropanoic acid	HMDB
107	107.038	C_3_H_6_O_4_ ° H+	Glyceric acid	HMDB
117	117.054 117.091	C_5_H_8_O_3_ ° H+ C_6_H_12_O_2_ ° H+	5‐Oxopentanoic acid Alpha‐ketoisovaleric acid Acetoxyacetone Caproic acid Ethyl 2‐methylpropanoate 2‐Methylpropyl acetate Isopropyl propionate Methyl (S)‐2‐methylbutyrate	HMDB HMDB HMDB
122	122.061 122.104	C_7_H_7_NO ° H+ C_8_H_11_N ° H+	3‐Acetylpyridine (1/2)‐Phenethylamine 5‐Ethyl‐2‐methylpyridine 2‐Propylpyridine 2‐Phenylethanaminium	HMDB HMDB

Results of the PTR‐MS (intervention 2) and PTR‐QiToF‐MS (intervention 3) were compared, *m*/*z* values were converted to molecular formulas using Chemcalc.^19^ Potential compounds were tentatively identified using the Human Metabolome Database (HMDB)^20^ and literature (references provided).

### 3.6 VOCs in Milk

**Figure 7:**
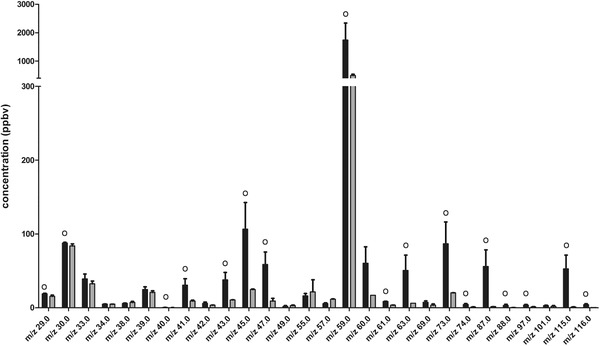
VOCs from the headspace of the high‐fat and low‐fat dairy drinks (mean ± SD) (black bars represent the high‐fat dairy drink and the grey bars represent the low‐fat dairy drink, °difference between the two dairy drinks was larger than 2 × SD).

VOCs in the headspace of the high‐fat and low‐fat dairy drinks were analyzed (**Figure** 7). A concentration above 0.2 ppbv was found for 28 *m*/*z* values in both drinks. Almost all of these *m*/*z* values were present in higher concentrations in the headspace of the high‐fat dairy drink compared to the low‐fat drink.

## 4 Discussion

This study showed that nutrition altered the exhaled VOC profile and that the consumption of two dairy drinks with a different fat percentage resulted in a different postprandial VOC profile. This implies that analysis of VOCs in exhaled air can be used to noninvasively assess responses to nutritional interventions. However, there was substantial variation between and within subjects, which needs to be taken into account for study design and data analysis.

### 4.1 Interindividual Variation

In this study, we observed high interindividual variation for VOC profiles, which is in line with previous findings.^3^ We attempted to minimize interindividual variation by including only males within a specific age and BMI range, since it was recently shown that BMI, gender, and age influence the VOC profile.^12^ However, we still found substantial differences in the VOC profiles of the different participants of intervention 1. Using preprocessing, we were able to remove a substantial portion of this variation.

### 4.2 Intraindividual Variation

Although the total postprandial VOC profile was similar on three different study days, we observed intraindividual variation as for some *m*/*z* values, the three study days differed quantitatively. *m*/*z* 69 is an example, which showed significant differences between study days, independent of the intervention. In accordance with previous literature,^21^, ^22^ this indicates that there is day‐to‐day variation in VOC concentrations. So, despite exclusion of alcohol consumption and use of drugs and standardization of exercise and dinner the day preceding a study day as well as standardization of the execution of the measurements, we still found intra‐subject variation. The source of this variation is still unknown. For our second intervention, multiple repetitions of each treatment were included as a solution. This, however, is not optimal, since the method was intended to reduce the burden for the participants.

### 4.3 Effect of Nutrition

This study showed a clear effect of nutrition on the VOC profile. From the second intervention, it can be concluded that consumption of two drinks differing only in the amount of fat resulted in a different VOC profile; about 23% of the total variation in the dataset can be explained by the difference in lipid content. Some individual VOCs were found to contribute to these different profiles, as different time responses after consumption of the two different drinks were found. Intervention 1 also clearly showed an effect of nutrition on some specific *m*/*z* values. For these *m*/*z* values, different profiles with time could be identified. These may be derived from different metabolic pathways. Some VOCs, such as *m*/*z* 45, showed an increase directly after consumption of the milk. These likely are compounds related to aroma/flavor in the milk, which are consumed and directly exhaled. Indeed, *m*/*z* 45 is also found in the headspace of the high‐fat milk.

### 4.4 Effect of Lipid Ingestion

The amount of ingested fat clearly affected the profile of VOCs. This could reflect effects on substrate metabolism, as a fat load was shown to profoundly influence substrate utilization.^23^ This was further supported by the tentative identification of the affected VOCs. *m*/*z* 59 was identified as acetone, and since *m*/*z* 60 and *m*/*z* 77 showed a very similar pattern with time, these most likely were an isotope and a water cluster of acetone, respectively, as was also suggested by Herbig et al. (2009).^24^ The strong increase 3 h after the consumption of the dairy drinks in acetone and related compounds suggested an increase in ketogenesis, which is in agreement with previous research.^25^ An increase in breath acetone has been related to an increase in fat oxidation.^26^ A previous study showed that fat oxidation increased above fasting levels 150 min after consumption of a high‐fat meal.^27^ So, it is very likely that in the present study, fat oxidation started to increase 180 min after the consumption of the dairy drinks. Since *m*/*z* 87 displayed a similar response curve as *m*/*z* 59, it is likely also a member of the ketone family, such as 2‐pentanone (Table 5). This ketone was, just like acetone, present in higher concentrations in exhaled air compared to ambient air,^28^ and was also found to be increased in fasting subjects.^29^ It is hypothesized to be formed during beta‐oxidation of caproic acid, at least in *Penicillium roqueforti*.^29^ Whether this process also occurs in humans is not known yet. The early increase in ketones after the consumption of the high‐fat drink might come from VOCs present in the milk, being immediately exhaled upon the diary drink intake. Indeed, *m*/*z* 59, *m*/*z* 60, and *m*/*z* 87 were present in higher concentrations in full‐fat milk compared to skimmed milk, as were some key butter aroma compounds.^30^

After the consumption of the high‐fat drink, an increase in *m*/*z* 107 was found, while this *m*/*z* value decreased after consumption of the low‐fat drink. This compound might have been glyceric acid; an oxidation product of glycerol. It was previously shown that consumption of dietary fat increases fat oxidation directly,^27^ the increase of glyceric acid might be a reflection of that. Another VOC which might be related to fat oxidation is *m*/*z* 56, which is possibly a fragment of hexanal. Hexanal is a breakdown product of linoleic acid oxidation. Surprisingly, *m*/*z* 56 decreased after consumption of the drinks, while it was expected that fat oxidation would increase after this high‐fat intake. The slight decrease may be explained by low levels of linoleic acid in bovine milk fat,^31^ providing a relatively lower amount of substrate that is oxidized into hexanal. *m*/*z* 117 might have been caproic acid (C6:0), a (combination of) short fatty acid ethyl ester(s), or metabolites related to amino acid degradation. Since participants were not allowed to consume alcohol on the day before the intervention, it is more likely that *m*/*z* 177 corresponds to caproic acid or metabolites related to amino acid degradation were exhaled, rather than to ethyl esters. Caproic acid is one of the medium‐chain fatty acids present in milk fat.^31^ This fatty acid is released during digestion in the stomach, and is very volatile. Potentially part of this fatty acid might have been exhaled before it could be absorbed. However, the increase in *m*/*z* 117 after the high‐fat and the low‐fat milk was similar. Since the low‐fat milk contained much lower concentrations of caproic acid, this is therefore a less likely explanation. Since *m*/*z* 117 showed a direct peak after consumption of the milk, it might also have been an aroma/flavor compound present in milk fat. However, as *m*/*z* 117 was not detected in the headspace of the milk products, it should then be released after digestion. The other *m*/*z* values were not identified or were difficult to link to metabolism. Altogether, this study showed that when nutrition influences metabolism, this might also be represented as VOCs in exhaled air.

### 4.5 VOCs in Nutrition Studies

Due to the great potential in clinical diagnostics and exposure assessment, analysis of VOCs in exhaled air has attracted a great deal of attention in the latest years.^1^ Especially for vulnerable target groups, this seems a promising method. In a recent study, GC‐MS was used to analyze VOCs patterns in exhaled air after consumption of two different infant formula products.^10^ Eight VOCs were significant different, only at the final time point, that is, 240 min after consumption of the products.^10^ In that study, participants received both treatments only once, so, based on our study, a considerable intra‐subject variation may have prevented identification of additional differentially affected VOCs. Although exhaled air is less complex than urine or blood samples, standardization and reproducibility have proven to be more difficult.^11^ This present study gave some additional insights in the inter‐ and intra‐subject variation. For now, some of this variation can be corrected for by choosing proper study designs, such as a cross‐over design and multiple repetitions, and preprocessing of the data. Further research could focus on how to minimize intra‐ and interindividual variation, for example, by standardizing exercise on a study day, by similar transportation to the study site, and by controlling exercise on multiple days before a study day. Since microbiota also produce VOCs,^14^ perhaps the diet, and therefore substrate for microbiota, on the whole day or several days before a study day could be controlled for. This present study showed that although these types of variation are present, the effect of nutrition on the VOC profile could clearly be assessed. A next step would be to relate VOCs in exhaled air with metabolites in blood samples, to verify whether these compounds are endogenous and to examine the link with metabolism in order to study whether VOCs can be used as biomarkers to study metabolic outcomes.

## 5 Conclusions

This study showed that nutrition, despite a considerable inter‐and intraindividual variability, had a strong influence on the post‐prandial exhaled VOC profile. Increasing the fat load affected specific VOCs that, based on their *m*/*z* values, are likely to be produced by metabolic pathways related to fat oxidation, such as ketogenesis and glycerol oxidation. Therefore, the analysis of VOCs can be considered a promising noninvasive method to study energy metabolism, which should be further explored for use in nutritional intervention studies. High interindividual variation still complicated VOCs analysis and may be controlled for by choosing a right study design and data processing. Important next steps to move this promising new technology toward practical application, are studies on how to reduce intraindividual variability, to link exhaled VOCs with biomarkers in blood, and to connect VOC responses to metabolic outcomes. Nevertheless, the information provided can to help to understand sources of variation in VOC analyses aiming at other goals, such as diagnosis of disease. By showing that VOCs in exhaled air can be used to discriminate between intake of drinks differing only in the amount of fat, we exemplify its potential use as a noninvasive tool in nutritional assessment.

## Conflict of Interest

J.H.J.H. is an employee of FrieslandCampina. Other authors declare no conflict of interest.

## Supporting information

Supporting Information

Supporting Figure

Supporting Figure

Supporting Figure

Supporting Figure
