# Supplementary material for: Application of Volatile Organic Compound Analysis in a Nutritional Intervention Study: Differential Responses during Five Hours Following Consumption of a High‐ and a Low‐Fat Dairy Drink
Source: Mol Nutr Food Res. 2019 Aug 5;63(20):1900189. doi: 10.1002/mnfr.201900189 (PMC6852046; doi:10.1002/mnfr.201900189)
Supplement: Supplementary file 1 — Supporting Information [file MNFR-63-na-s005.docx]

**Supporting information**

**Figure 1. Overview of segmented regression lines of VOCs measured in exhaled air after consumption of high-fat and low-fat dairy drinks, from 15 – 300 minutes postprandial.** The green lines represent the high-fat dairy drink and the red lines represent the low-fat dairy drink, averages of 12 subjects are shown.
