# Supplementary figures and images for: Application of Volatile Organic Compound Analysis in a Nutritional Intervention Study: Differential Responses during Five Hours Following Consumption of a High‐ and a Low‐Fat Dairy Drink
Source: Mol Nutr Food Res. 2019 Aug 5;63(20):1900189. doi: 10.1002/mnfr.201900189 (PMC6852046; doi:10.1002/mnfr.201900189)

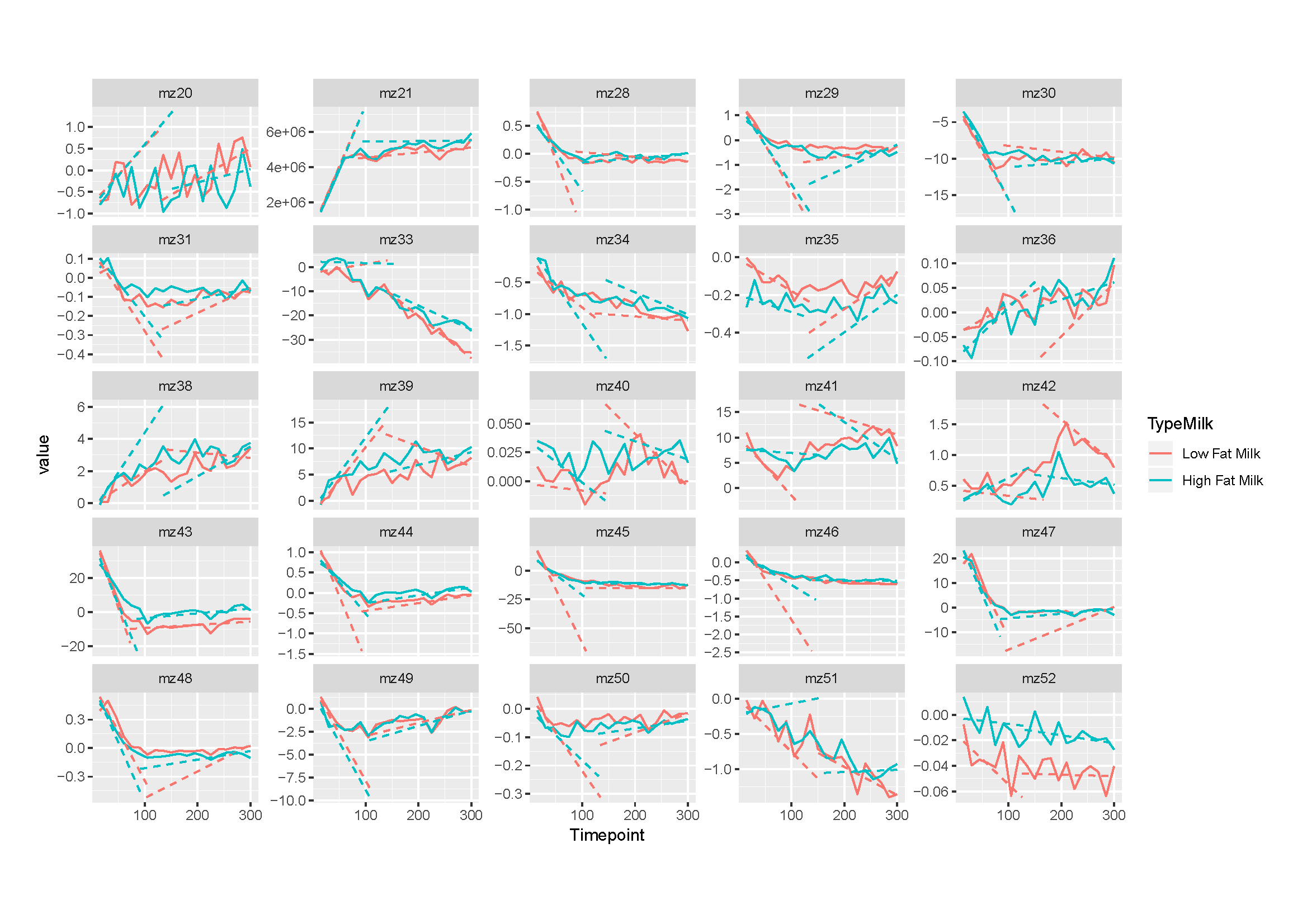

Supplement: Supplementary file 2 — Supporting Figure [file MNFR-63-na-s001.tiff]

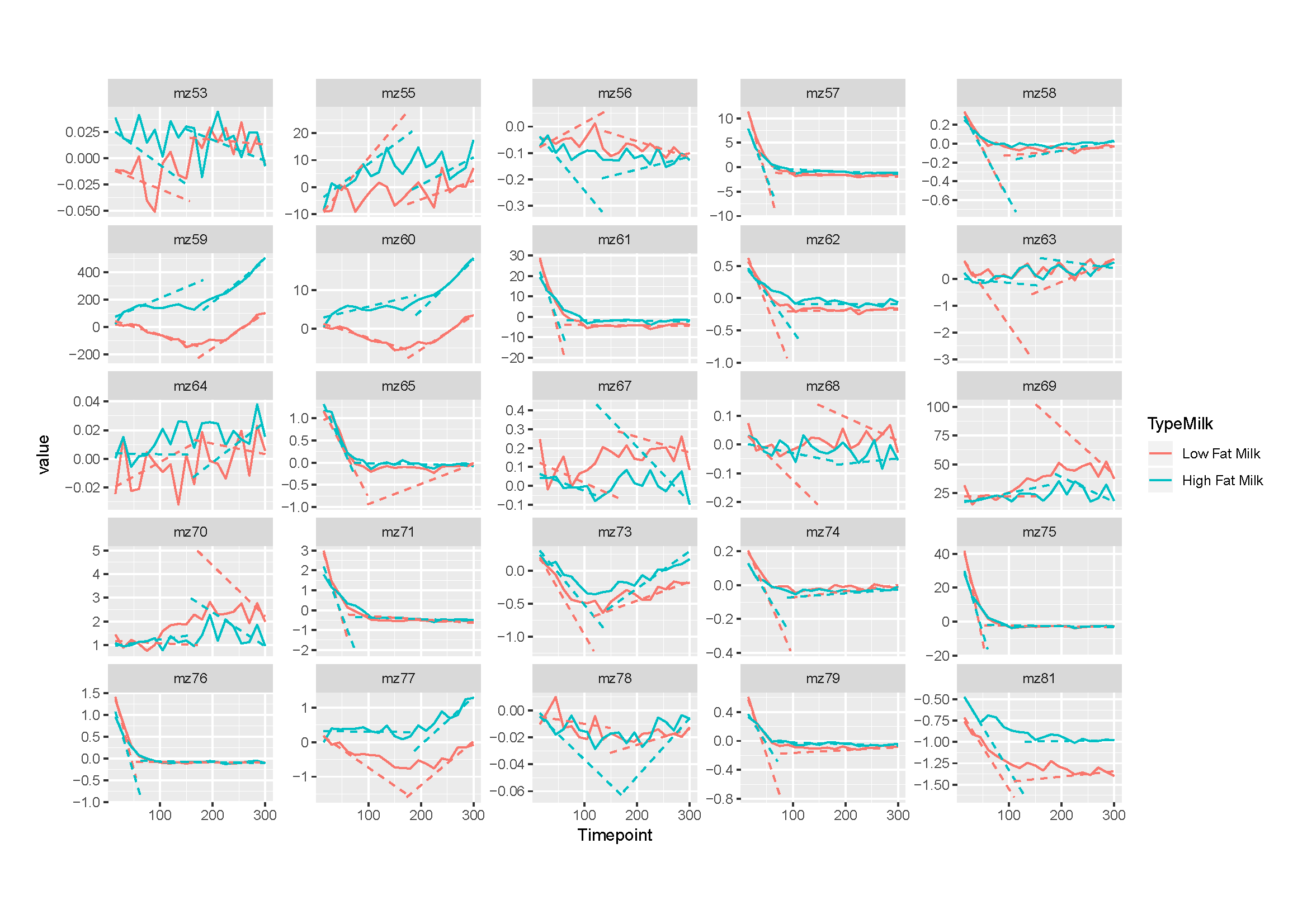

Supplement: Supplementary file 3 — Supporting Figure [file MNFR-63-na-s002.tiff]

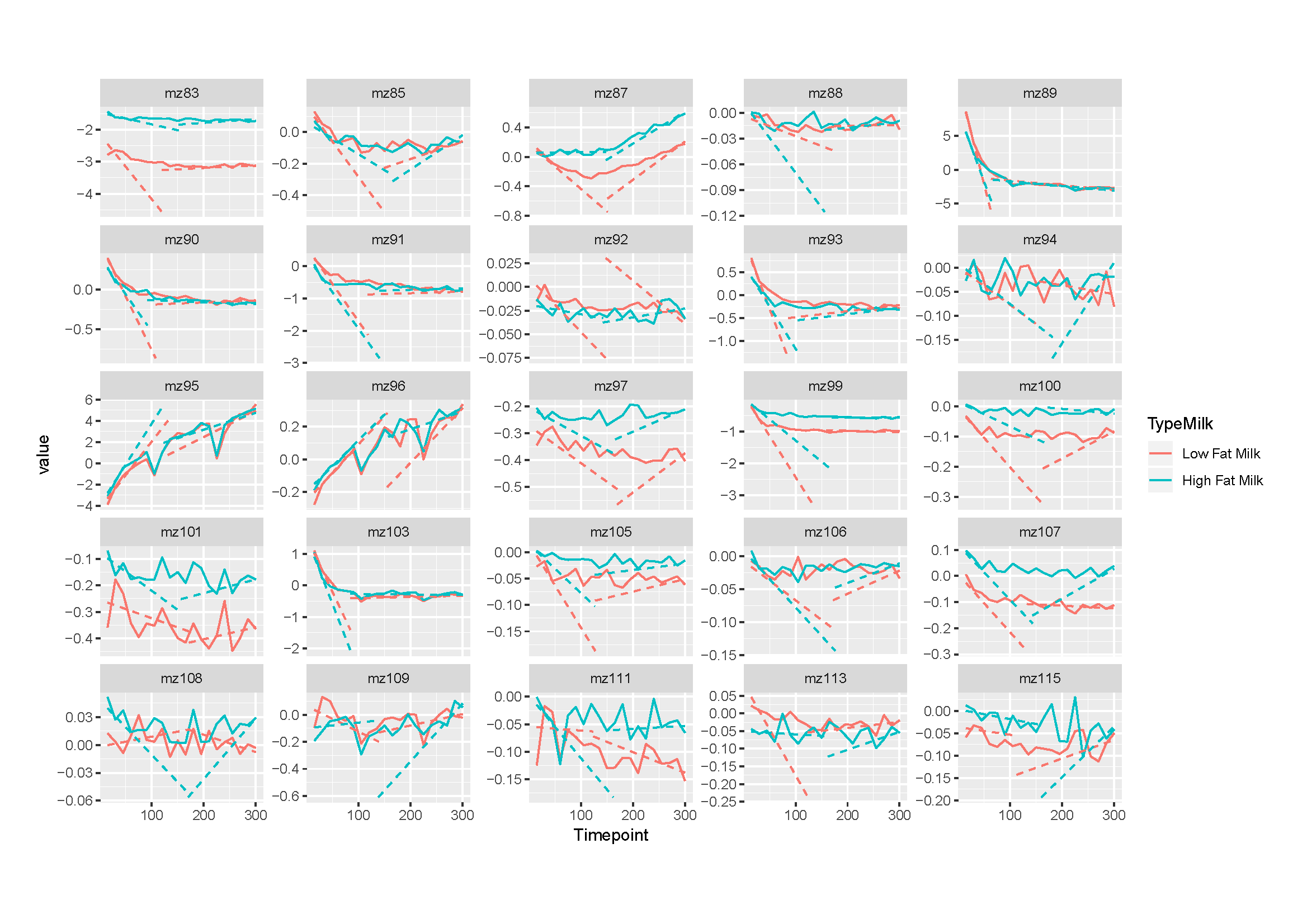

Supplement: Supplementary file 4 — Supporting Figure [file MNFR-63-na-s003.tiff]

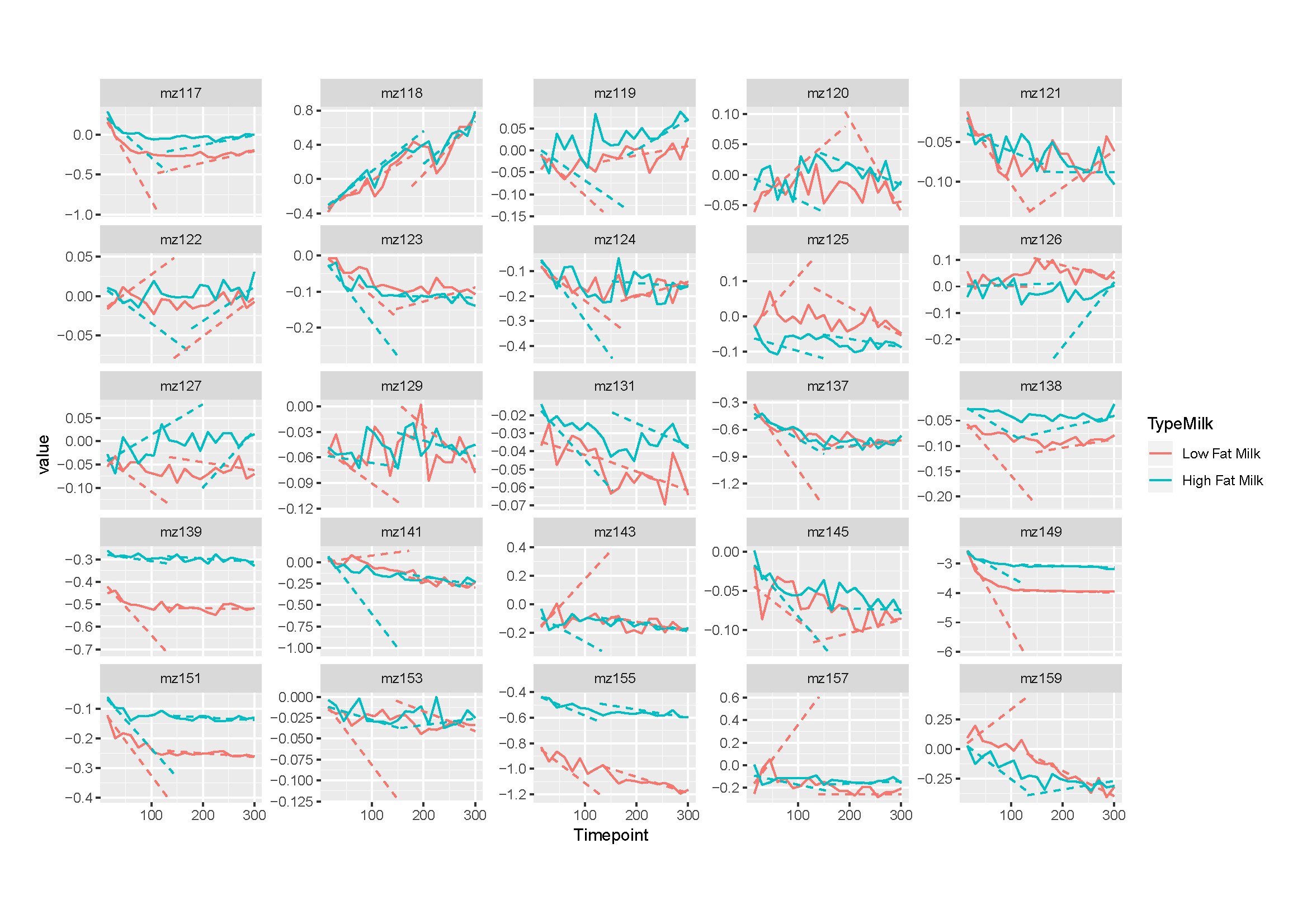

Supplement: Supplementary file 5 — Supporting Figure [file MNFR-63-na-s004.tiff]
